# PEGylated Amine-Functionalized Poly(ε-caprolactone) for the Delivery of Plasmid DNA

**DOI:** 10.3390/ma13040898

**Published:** 2020-02-18

**Authors:** Amin Jafari, Nika Rajabian, Guojian Zhang, Mohamed Alaa Mohamed, Pedro Lei, Stelios T. Andreadis, Blaine A. Pfeifer, Chong Cheng

**Affiliations:** 1 Department of Chemical and Biological Engineering, University at Buffalo, The State University of New York, Buffalo, NY 14260, USA; aminjafa@buffalo.edu (A.J.); nikaraja@buffalo.edu (N.R.); zhangguojian@ouc.edu.cn (G.Z.); mm446@buffalo.edu (M.A.M.); pedrolei@buffalo.edu (P.L.); sandread@buffalo.edu (S.T.A.); blainepf@buffalo.edu (B.A.P.); 2 Key Laboratory of Marine Drugs, Chinese Ministry of Education, School of Medicine and Pharmacy, Ocean University of China, Qingdao 266003, China; 3 Laboratory for Marine Drugs and Bioproducts of Qingdao National Laboratory for Marine Science and Technology, Qingdao 266237, China; 4 Chemistry Department, Faculty of Science, Mansoura University, Mansoura 35516, Egypt

**Keywords:** Non-viral gene delivery, cationic polymer, poly(ε-caprolactone), poly(ethylene glycol), ring-opening polymerization (ROP), polyplex, nanocomplex, plasmid DNA (pDNA), PEGylation

## Abstract

As a promising strategy for the treatment of various diseases, gene therapy has attracted increasing attention over the past decade. Among various gene delivery approaches, non-viral vectors made of synthetic biomaterials have shown significant potential. Due to their synthetic nature, non-viral vectors can have tunable structures and properties by using various building units. In particular, they can offer advantages over viral vectors with respect to biosafety and cytotoxicity. In this study, a well-defined poly(ethylene glycol)-*block*-poly(α-(propylthio-*N*,*N*-diethylethanamine hydrochloride)-ε-caprolactone) diblock polymer (PEG-*b*-CPCL) with one poly(ethylene glycol) (PEG) block and one tertiary amine-functionalized cationic poly(ε-caprolactone) (CPCL) block, as a novel non-viral vector in the delivery of plasmid DNA (pDNA), was synthesized and studied. Despite having a degradable polymeric structure, the polymer showed remarkable hydrolytic stability over multiple weeks. The optimal ratio of the polymer to pDNA for nanocomplex formation, pDNA release from the nanocomplex with the presence of heparin, and serum stability of the nanocomplex were probed through gel electrophoresis. Nanostructure of the nanocomplexes was characterized by DLS and TEM imaging. Relative to CPCL homopolymers, PEG-*b*-CPCL led to better solubility over a wide range of pH. Overall, this work demonstrates that PEG-*b*-CPCL possesses a range of valuable properties as a promising synthetic vector for pDNA delivery.

## 1. Introduction

The first clinical study on gene therapy was conducted at the National Institutes of Health (NIH) in 1989, and it demonstrated that genetically modified human cells could be transferred to patients in a feasible and safe manner [1]. Following this study, almost 2600 clinical trials on gene therapy have been initiated or performed worldwide through November 2017 [2]. Thus far, six gene delivery products have been approved by the U.S. Food and Drug Administration (FDA), European Marketing Authorization (EMA), and the China Food and Drug Administration (CFDA), indicating steady development and growing confidence in gene therapy [2].

Nucleic acid, as the building block of genes, is highly polar, incapable of diffusing through cell membranes effectively [3]. Moreover, DNA therapeutics can undergo degradation by endonucleases in physiological fluids and the extracellular space [4]. Therefore, various approaches for gene transfer—such as physical, biological (viral vectors), and biomaterial (non-viral vectors) transfection methods—have been developed [3,5,6]. Physical methods involve the direct insertion of genes into the cells and can be categorized into mechanical approaches (microinjection [7] and particle bombardment [8,9]), electroporation [10], sonoporation [11], laser irradiation [12], and magnetofection [13]. Despite the safety and simplicity of genetic materials injections without any carrier, these technologies suffer mainly from fast clearance, rapid degradation by nucleases, and limited local distribution in tissue, although they can be improved by external means such as electric field and ultrasound positioning [5,14,15]. Extensively studied viral vectors as biological carriers for genetic materials, in spite of having suitable transfection efficiencies, can cause serious concerns including cytotoxicity, cargo capacity, immunogenicity, and difficulty in vector scale-up [16,17]. Concerns related to viral vectors may be circumvented by the exploitation of non-viral biomaterials as the delivery vectors. However, non-viral vectors have shown lower transfection efficiencies compared to viral vectors, which have spurred researchers to optimize their design [4]. Non-viral vectors can be categorized into lipids, cationic polymers, peptides, nucleic acid, and inorganic nanoparticles [4,18].

Among the non-viral vectors, cationic polymers (synthetic and natural) have been actively studied [19]. Given the negative charge of therapeutic nucleic acid-based macromolecules, their systemic delivery was achieved through electrostatic interactions with cationic polymers to form spherical nanoparticles, which are termed polyplexes or nanocomplexes [4]. The polyplex envelops therapeutic DNA, protects nucleic acid cargo from endonuclease degradation, and improves in vivo circulation time [4]. Versatile formulation of polymeric structures with post-polymerization modifications allows polyplexes to be tuned so that their enhanced gene delivery may approach the ideal case of viral vectors. Poly(L-lysine) (PLL) and polyethyleneimine (PEI) are among the early and mostly studied cationic polymeric DNA vectors for gene delivery [4]. PEIs with various molecular weight (MW) and non-degradable structures (linear or branched) can exhibit different transfection efficiencies and toxicity [19]. Naturally-derived cationic polymers, such as chitosan and modified gelatin, have been studied for gene delivery, but the corresponding transfection efficiencies are typically quite limited [20].

Among various cationic polymers, we are particularly interested in cationic aliphatic polyesters as novel biodegradable non-viral vectors for gene delivery applications [21,22,23,24]. Our previous studies on tertiary amine-functionalized cationic polylactides (CPLAs) showed effectiveness as vectors for the delivery of pDNA and small interfering ribonucleic acid (siRNA) [25,26,27,28,29]. Relative to CPLA homopolymers, well-defined poly(ethylene glycol)-*block*-cationic polylactide (PEG-*b*-CPLA) diblock copolymers can further lead to high levels of transfection and serum stability with low levels of cytotoxicity and hemolysis [25]. Moreover, it was demonstrated that the CPLA-based nanocapsules are effective vectors for individual delivery and co-delivery of genes and drugs to cancer cells [28]. The aforementioned merits of CPLAs and the derived biomaterials notwithstanding, they have a relatively short shelf life in aqueous solutions due to their low hydrolytic stability, which restricts their transformative potential in biomedical applications [26,28].

**Figure 1:**
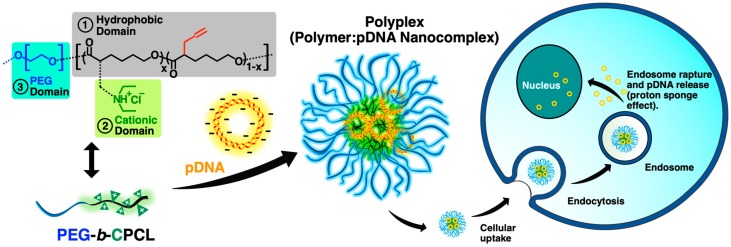
Non-viral pDNA delivery using PEG-*b*-CPCL.

In this article, we report upon poly(ethylene glycol)-*block*-poly(α-(propylthio-*N*,*N*-diethylethanamine hydrochloride)-ε-caprolactone) diblock polymer (PEG-*b*-CPCL) with one poly(ethylene glycol) (PEG) block and one tertiary amine-functionalized cationic poly(ε-caprolactone) (CPCL) block (Figure 1), as a novel potential vector for the delivery of pDNA. This well-defined diblock polymer is prepared by ring-opening polymerization (ROP) and features tertiary amine-functionalized cationic groups carried by the biodegradable poly(ε-caprolactone) (PCL) backbone. The existence of the ester group on the PCL backbone ensures biodegradability and promotes the eventual elimination of the polymer vector for in vivo applications. Because PCL has a lower density of ester groups than polylactide (PLA), the CPCL-based polymer is expected to have higher hydrolytic stability than a CPLA-based polymer. Conjugation of cationic tertiary amine groups on the hydrophobic PCL block of PEG-*b*-CPCL enables the polymer to form polyplexes with genetic material, mainly in the CPCL-based interior domain of the polyplex nanostructure while protecting nucleic acid cargo from degradation by serum endonucleases and evading immune detection. Moreover, because the tertiary amine groups of CPCL block can adsorb protons within endosomes to induce proton sponge effect, the intracellular release of polyplexes after endocytosis can be facilitated [26]. On the other hand, the hydrophilic PEG block of the PEG-*b*-CPCL diblock copolymer results in a PEG-based exterior shell domain of the polyplex nanostructure, leading to improved aqueous solubility and colloidal stability as well as increased circulation time of the polyplexes through minimizing nonspecific interactions with serum components. Figure 1 demonstrates how PEG-*b*-CPCL forms polyplexes with pDNA and facilitates the transfection process upon administration.

## 2. Materials and Methods

### 2.1. Measurements

^1^H NMR measurements were conducted at 500 MHz using a Varian INOVA-500 (Varian, Inc., Palo Alto, CA, USA). The temperature of all measurements were set at 25 °C and tetramethylsilane (TMS) was used as an internal reference. VnmrJ software (v. 4.2, Agilent Technologies, Inc., Santa Clara, CA, USA) with a university license was used to analyze the raw data.

The analysis of polymer MW and MW dispersity (*Đ*) was conducted using gel permeation chromatography (GPC) technique. The instrument was a Viscotek GPC system (Malvern Panalytical, Malvern, UK) having a VE-1122 pump, a VE-3580 refractive index (RI) detector, and two mixed-bed organic columns (PAS-105M and PAS-103M). The GPC instrument was operated with dimethylformamide (DMF; HPLC; with 0.01 M LiBr salt) as the mobile phase at a flow rate of 0.5 mL/min at 55 °C. The calibration was conducted by using linear polystyrene standards purchased from Varian (Varian, Inc., Palo Alto, CA, USA); peak MW (*M*_p_) = 0.58, 1.53, 3.95, 10.21, 29.51, 72.45, 205, 467, 1319, and 2851 kDa).

Hydrodynamic diameter (*D*_h_) and zeta potential (ζ) of polymer:pDNA nanocomplexes were analyzed by using dynamic light scattering (DLS). The instrument was a Zetasizer nano-ZS90 (Malvern Panalytical, Malvern, UK), with a 4 mW 633 nm HeNe laser as the light source. All experiments were conducted at a fixed measuring angle of 90° to the incident laser beam and 25 °C. Zetasizer Nano software (v. 7.11, Malvern Panalytical, Malvern, UK) was used to analyze the raw data for both *D*_h_ and ζ.

Transmission electron microscopy (TEM) images were taken using a JEOL 2010 microscope (JEOL Ltd., Akishima, Japan). TEM samples were prepared using 400 mesh carbon-coated copper grids. Sample solutions with DLS count rate of ~250 kcps in water were dip coated onto the TEM grids. Then the water was completely dried under vacuum and no staining was applied for the samples.

### 2.2. Materials

1,5,7-Triazabicyclo[4.4.0]dec-5-ene (TBD), methoxy poly(ethylene glycol) (mPEG_45_-OH; *M*_n_ ~ 2000 Da, flakes), and hexamethylphosphoramide (HMPA; 99%) were purchased from Sigma-Aldrich (Saint Louis, MO, USA). ε-Caprolactone (CL) monomer (99%), propargyl bromide (80 wt.% solution in toluene, stabilized), lithium diisopropylamide (LDA; 2M solution in THF/*n*-hexane/ethylbenzene, nitrogen flushed), lithium bromide (LiBr; 99+%, anhydrous), sodium chloride (99+%), benzyl alcohol (BnOH; 99%), nile red (99%), and 2,2′-dimethoxy-2-phenylacetophenone (DMPA; 98%) were purchased from Acros Organics (Geel, Belgium). Magnesium sulfate (99%, anhydrous), toluene (Certified ACS), dichloromethane (DCM; HPLC), chloroform (HPLC), acetone (HPLC), tetrahydrofuran (THF, HPLC), ethyl acetate (EA; HPLC), *N*,*N′*-dimethylformamide (DMF, HPLC), and diethyl ether (HPLC) were purchased from Fisher Scientific (Waltham, Massachusetts, MA, UWA). 2-Diethylaminoethanethiol hydrochloride (DEAET, >98%) was purchased from Amfinecom Inc. (Petersburg, VA, USA) Heparin sodium salt from porcine intestinal mucosa (Grade I-A, ≥180 USP units/mg) was purchased from Sigma-Aldrich (Saint Louis, MO, USA).

Toluene was refluxed over CaH_2_ for 2 h prior to distillation. THF was dried by distillation over sodium-benzophenone when the solution was dark blue. Water content of mPEG_45_-OH was removed by two times of azeotropic distillation in dry toluene at 175 °C. HMPA and CL were distilled from CaH_2_ shortly before use. LDA, as a pyrophoric material that is highly flammable, water reactive, toxic, corrosive and known to be carcinogenic and teratogenic, was used according to strict safety guidelines. Deionized water, obtained from a Thermo Scientific Barnstead Nanopure ultra-pure water system, was used for the preparation of all buffer solutions. All other chemicals were used without further purification.

### 2.3. Synthesis of Allyl-Functionalized CL (ACL, ***1***)

**Scheme 1:**
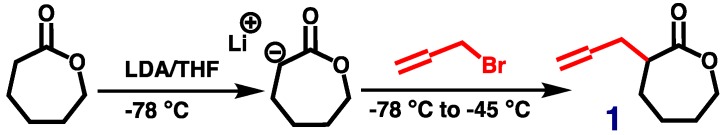
Synthesis of allyl-functionalized CL (ACL; **1**).

**Figure 2:**
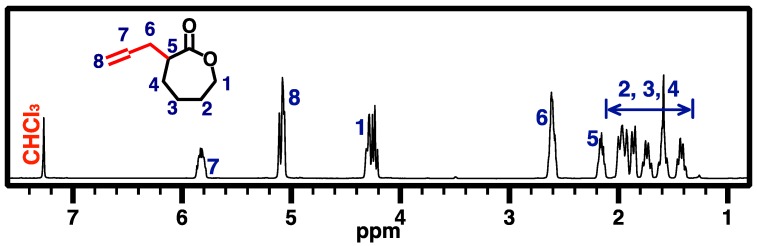
500 MHz ^1^H NMR spectrum of ACL (**1**) in CDCl_3_.

ACL **1** was synthesized according to a previously reported procedure (Scheme 1) [30]. Briefly, 150 mL of freshly distilled dried THF was air-free transferred to a vacuum dried 250-mL reaction flask with a magnetic stirring bar under vigorous nitrogen purging and cooled in a dry ice/acetone bath. Upon reaching a solution temperature of −78 °C, the LDA solution (2.0 M, 25 mL) was carefully added dropwise to the reaction flask. After stirring for a few minutes, a diluted solution of CL (5.00 g) in THF (30 mL) was added slowly over a period of 1 h. This mixture was stirred at −78 °C for another 1 h, and allyl bromide (6 mL) in HMPA (10 mL) was added dropwise. The reaction system was allowed to warm up over 1 h to −45 °C and was kept at this temperature for another 1.5 h. The reaction was quenched by adding a saturated NH_4_Cl solution to the flask. The reaction mixture was allowed to warm up to room temperature and then washed with saturated NaCl and NH_4_Cl solutions (3×). The upper organic layer was concentrated and added to cold diethyl ether. The white precipitate was removed by filtration, and the filtrate was dried over magnesium sulfate and concentrated under vacuum. The resulting crude product was purified through a silica gel column chromatography eluted with hexane/EA (7/3) to give the desired product **1** (2.43 g, 36% yield; R_f_ = 0.35) in the form of a very light-yellow clear oil. Its chemical structure was confirmed by ^1^H NMR analysis in CDCl_3_ (Figure 2).

### 2.4. Synthesis of PEG-b-(Allyl-Functionalized PCL) (PEG-b-APCL, ***2***)

**Figure 3:**
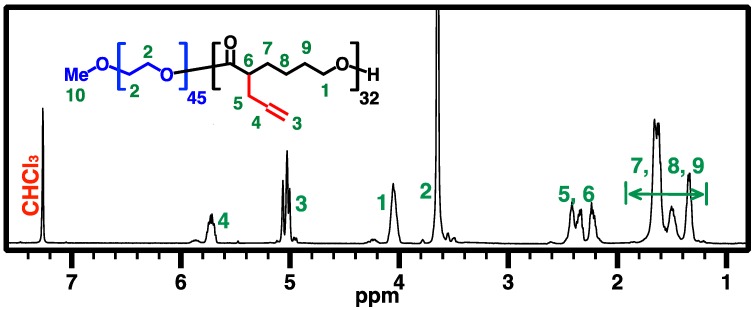
500 MHz ^1^H NMR spectrum of PEG-*b*-APCL (**2**) in CDCl_3_.

PEG-*b*-APCL **2** was synthesized based on a literature method of ring-opening polymerization (ROP) of ACL **1** using TBD as organocatalyst [31,32]. A 10-mL Schlenk flask with a magnetic stirring bar was flamed dried under vacuum and refilled with nitrogen (3×). Preweighted amount of dried mPEG_45_-OH macroinitiator (65.69 mg, 33 μmol, 1.0 equiv.) and dry TBD (22.86 mg, 164 μmol, 5.0 equiv.) were dissolved in dry toluene (2.5 mL), and then added to the flask via nitrogen-purged syringe. The solution was stirred for 30 min at room temperature. ACL monomer **1** (500 mg, 3.28 mmol, 100 equiv.) was dissolved in dry toluene (2.5 mL) and added to the flask with an air-free syringe to start ROP at room temperature. GPC and ^1^H NMR characterizations were used to monitor the ROP process and monomer conversion, respectively. Once the desired monomer conversion was achieved after 19 h, the reaction was quenched by adding several drops of acetic acid. The polymer was isolated by precipitation in cold diethyl ether (3×) to obtain a beige pasty solid (150 mg, conversion 32%, yield 94%). Figure 3 shows the 500 MHz ^1^H NMR spectrum of PEG-*b*-APCL (**2**) in CDCl_3_.

### 2.5. Synthesis of PEG-b-CPCL-70 (***3***)

**Figure 4:**
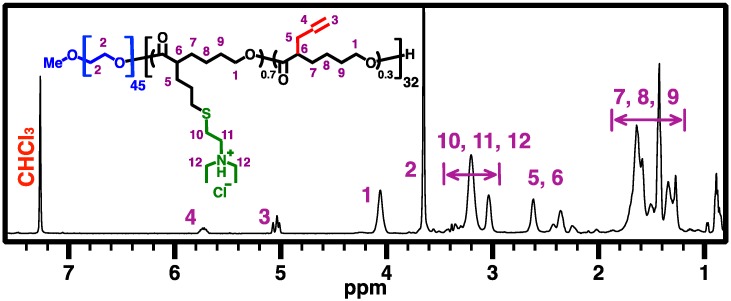
500 MHz ^1^H NMR spectrum of PEG-*b*-CPCL-70 (**3**) in CDCl_3_.

In a 10-mL Schlenk flask with a magnetic stirring bar, PEG-*b*-PCL (**2**) (150 mg, 705 μmol of allyl group) was dissolved in 5 mL anhydrous chloroform. DEAET (110 mg, 756 μmol, 1.1 equiv. relative to allyl group) and DMPA (36 mg, 141 μmol, 0.2 equiv. relative to allyl group) were added to the reaction flask. The reaction flask was sealed carefully, and the reaction mixture was degassed by five freeze-pump-thaw cycles, followed by irradiation with UV light (λ_max_ = 365 nm) for 40 min. Then the reaction mixture was dialyzed against DCM for 4 days (MW cut-off (MWCO) = 3.5 kDa) and dried under vacuum to obtain PEG-*b*-CPCL-70 (**3**, the number 70 indicating mole percent of amine group relative to CL unit) as a white pasty solid (166 mg, 76% isolated yield). Figure 4 shows 500 MHz ^1^H NMR spectrum of PEG-*b*-CPCL-70 (**3**) in CDCl_3_.

### 2.6. Synthesis of Allyl-Functionalized Poly(*ε*-caprolactone) (APCL) (***4***)

**Figure 5:**
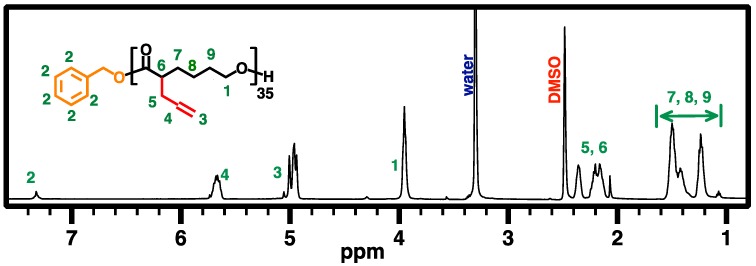
500 MHz ^1^H NMR spectrum of APCL (**4**) in DMSO-d_6_.

Based on a literature method of ring-opening polymerization (ROP) of ACL **1** using TBD as an organocatalyst [31,32], APCL (**4**) was synthesized. In a 10-mL flamed dried Schlenk flask with a magnetic stirring bar under nitrogen atmosphere, TBD (45 mg, 324 μmol, 3.5 equiv.) was dissolved in dry toluene (5.0 mL), followed by the addition of BnOH (10 mg, 92.6 μmol in a stock solution in dry toluene, 1.0 equiv.). The mixture was then stirred for 30 min at room temperature under nitrogen protection. ACL monomer **1** (1.00 g, 6.50 mmol, 70 equiv.) was dissolved in dry toluene (5.0 mL) and added to the flask with an air-free syringe to start ROP at room temperature. ROP was allowed to continue until it reached desired monomer conversion, as observed by ^1^H NMR analysis. After 13.5 h, ROP was quenched by adding several drops of acetic acid. APCL was isolated by precipitation in cold methanol (3×), yielding a light-yellow viscous oil (333 mg, 50% conversion of **1**, 67% isolated yield). Figure 5 illustrates the 500 MHz ^1^H NMR spectrum of APCL (**4**) in DMSO-d_6_.

### 2.7. Synthesis of Cationic Poly(*ε*-caprolactone) (CPCL) (***5***)

**Figure 6:**
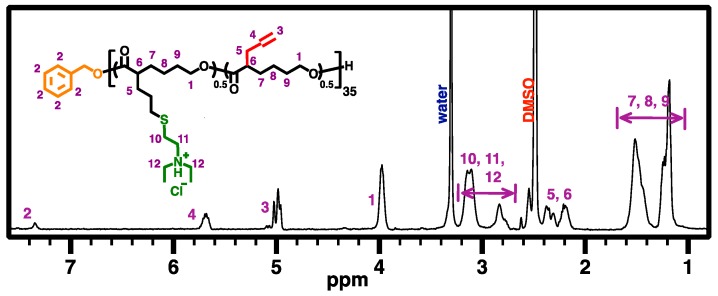
500 MHz ^1^H NMR spectrum of CPCL-50 in DMSO-d_6_.

For the synthesis of CPCL-50 and CPCL-95 (the numbers, i.e., 50 and 95, indicating mole percent of amine group relative to CL unit), in a 10-mL Schlenk flask with a magnetic stirring bar, APCL (**4**) (75.0 mg, 495 μmol of allyl group) was dissolved in 3.0 mL of anhydrous DCM. For the preparation of CPCL-50, DEAET (52 mg, 371 μmol, 0.75 equiv. relative to allyl group) and DMPA (25 mg, 99 μmol, 0.2 equiv. relative to allyl group) were added to the reaction flask. For the preparation of CPCL-95, DEAET (210 mg, 1.48 mmol, 3.0 equiv. relative to allyl group) and DMPA (50 mg, 198 μmol, 0.4 equiv. relative to allyl group) were used. The reaction flasks were sealed carefully, and the reaction mixtures were degassed by five freeze-pump-thaw cycles. They were irradiated by UV light (λ_max_ = 365 nm) for 1 h and 3 h to obtain CPCL-50 and CPCL-95, respectively. The reaction mixtures were then precipitated in hexane (3×) and dialyzed against DCM (MWCO = 3.5 kDa) for 4 days and vacuum dried to obtain pure products. CPCL-50 (89 mg, 50% conversion of allyl groups, 81% isolated yield) and CPCL-95 (125 mg, 95% conversion of allyl groups, 88% isolated yield) were in the form of light yellow and brittle white hygroscopic solids, respectively. Figure 6 illustrates the 500 MHz ^1^H NMR spectrum of CPCL-50 in DMSO-d_6_, which is similar to the spectrum of CPCL-95 (not shown).

### 2.8. Study of Hydrolytic Stability of PEG-b-CPCL-70 (***3***)

Two PBS buffer solutions of pH 5.5 and 7.4 at a concentration of 25 mM were prepared. PEG-*b*-CPCL-70 was dissolved in prepared buffer medium at a concentration of 1 mg/mL. The solutions were then transferred to separate sealed vials and incubated at 37 °C with mild shaking. At specific time intervals, aliquots from each vial were taken and completely dried under vacuum to remove any moisture. The resulting solid was dissolved in DMF (containing 0.01 M LiBr) and analyzed with GPC to assess hydrolytic stability.

### 2.9. Preparation of Polymer:pDNA Nanocomplexes

Nanocomplexes were formed based on spontaneous electrostatic interactions between PEG-*b*-CPCL-70 and pCMV-EGFP pDNA at various polymer:pDNA mass ratios. The pDNAs were produced by an *Escherichia coli* bacterial host (GeneHogs, Invitrogen), with an enhanced green fluorescent protein reporter gene, driven by a cytomegalovirus promoter within plasmids (pCMV-EGFP, Addgene). A stock aqueous solution of polymer was prepared at a concentration of 4 mg/mL in ultrapure water. For each polymer:pDNA nanocomplex mass ratio, a specific amount of polymer solution was mixed with a pDNA solution, gently vortexed, and incubated for 30 min to ensure the formation of polymer:pDNA nanocomplexes. For example, for the preparation of nanocomplex at 200:1 of PEG-*b*-CPCL-70:pDNA mass ratio, 400 μL of polymer stock solution (4 mg/mL) was mixed with pCMV-EGFP pDNA (with a final concentration of 8 µg/mL) to a final volume of 1 mL.

### 2.10. N/P Ratio Calculation

N/P ratio denotes the molecular ratio of cationic amine groups (N) in the PEG-*b*-CPCL-70 to anionic phosphate groups (P) in pDNA [33]. The mass per mole for one anionic charge (P) of pDNA is 330 g/mol [34], whereas the mass per mole for one cationic charge (N) of PEG-*b*-CPCL-70 is 465 g/mol. The value of 465 g/mol was obtained by dividing *M*_n_^NMR^ of PEG-*b*-CPCL-70 with the number of cationic amine groups (N) of the polymer. Therefore, in a PEG-*b*-CPCL-70:pDNA nanocomplex, the N/P ratio was calculated as:

(1) $$N/P=\frac{\left(\frac{\mu g\;\mathrm{of}\;\mathrm{polymer}}{465\;\left(g/\mathrm{mol}\right)}\right)}{\left(\frac{\mu g\;\mathrm{of}\;\mathrm{pDNA}}{330\;\left(g/\mathrm{mol}\right)}\right)}$$

This equation was used to convert PEG-*b*-CPCL-70:pDNA mass ratios to N/P ratios (N/P ratios = 0.71 mass ratios).

### 2.11. pDNA Gel-shift Assay

The polymer:pDNA nanocomplexes were tested with a gel electrophoresis assay. Samples of nanocomplexes were loaded into a 1% agarose gel with ethidium bromide staining and electrophoresed at 100 V for 30 min. Gels were visualized under UV after electrophoresis to evaluate the pDNA shift at various polymer:pDNA mass ratios for nanocomplex formation.

### 2.12. pDNA Release by Polyanionic Heparin

The binding of pDNA to the polymeric domain within the nanocomplex was examined using polyanionic heparin to release the pDNA [35]. Nanocomplexes were prepared at a polymer:pDNA mass ratio of 200:1 to guarantee the complete condensation of pDNA in the cationic polymer matrix, followed by incubation at 37 °C for 1 h. The samples with various nanocomplex:heparin mass ratios were run on an agarose gel electrophoresis system as described earlier.

### 2.13. Serum Stability of Polymer:pDNA Nanocomplexes

The stability of pDNA in PEG-*b*-CPCL-70:pDNA nanocomplexes against fetal bovine serum (FBS, Atlanta Biologicals) was studied by agarose gel electrophoresis [36]. A 300 μL solution of polymer:pDNA nanocomplexes at a 200:1 mass ratio was incubated with 10% FBS for 24 h. Aliquots of 25 μL at different time points were taken and frozen immediately. Prior to agarose gel electrophoresis, 4 μL (1 mg/mL) of polyanionic heparin solution was added to each aliquot to ensure the release of pDNA from nanocomplexes. Intact pDNA species were then assessed by comparing DNA fragmentation patterns.

## 3. Results and Discussion

### 3.1. Synthesis and Characterization of PEG-b-CPCL-70 (***3***) and CPCLs (***5***)

Aliphatic polyesters are among the most widely studied synthetic polymers for biomedical applications due to their remarkable biodegradability. In our previous studies, we investigated the delivery of genetic material, such as pDNA and siRNA, using CPLAs [25,26,27]. It was found that these PLA-based materials have promising transfection efficiency, but they suffer from fast hydrolytic degradation (i.e., they degrade in aqueous solutions within a week), which limits their practical application [25,27]. Besides PLA, PCL composed of hexanoate repeating units is also a predominant aliphatic polyester and has been utilized extensively in biomedical applications. PCL has a lower density of ester groups on its backbone, and, therefore, offers slower degradation under biologically relevant conditions in comparison to PLA. The degradation of PCL produces 6-hydroxyhexanoic acid as the eventual small-molecule degradation residue, which is a natural human body metabolite. Generally, PCL can biodegrade over a longer time span from several months to several years, depending on MW, degree of crystallinity, and the condition of degradation [37]. Moreover, relative to PLA, PCL provides a more hydrophobic domain, which is less accessible to water due to the presence of five hydrophobic methylene moieties on each repeating unit of PCL. Therefore, in this study, we investigated tertiary amine-based CPCL polymers for pDNA delivery. Specifically, PEGylated CPCL, i.e., PEG-*b*-CPCL, was the polymer of interest in this investigation because of the merit of PEGylated synthetic vectors, as revealed in our previous work [25]. Non-PEGylated CPCLs were used along with PEGylated CPCLs in buffer solubility tests.

Because polymer synthesis for advanced applications such as drug/gene delivery requires the control over the polymer MW, MW dispersity (*Đ*), and functional groups, synthetic routes of these CPCL-based polymers were designed with two key steps: (1) the living ROP of ACL **1** to obtain APCL-based precursor polymers, and (2) the subsequent post-polymerization transformation of allyl groups to tertiary amine groups via thiol-ene click functionalization. ACL **1** was prepared following an approach from the literature (Scheme 1) [30], with chemical structure verified by ^1^H NMR analysis in CDCl_3_ (Figure 2).

**Scheme 2:**
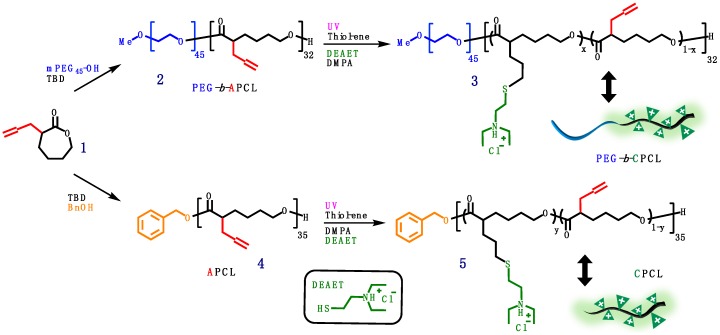
Synthesis of PEG-*b*-CPCL (**3**) and CPCL (**5**).

Scheme 2 illustrates the synthesis of PEGylated and non-PEGylated CPCLs. In the living ROP of **1**, TBD was used as the organocatalyst because it has relatively high catalytic efficacy that can result in dual activation of both monomer and alcoholic initiator [31], leading to the ROP process of CL monomer in a rapid and well-controlled manner (with a linear relationship between conversion and MW). The preparation of PEG-*b*-APCL, i.e., the diblock precursor of PEG-*b*-CPCL, was carried out in toluene at room temperature for 19 h by using mPEG_45_-OH (MW: 2000 Da; *Đ*^GPC^ = 1.03) as the macroinitiator for ROP of **1** ([M]_0_:[I]_0_:[TBD] = 100:1:5; ~32% conversion of **1**). The well-defined chemical structure of the resulting PEG_45_-*b*-APCL_32_ (i.e., **2**) was verified by 500 MHz ^1^H NMR analysis in CDCl_3_ (Figure 3). The number-average degree of polymerization (*DP*_n_) of 32 for the APCL block was determined based on a comparison of resonance intensity of C*H*_2_OCO protons of the APCL block centered at 4.04 ppm with that of C*H*_2_C*H*_2_O protons of the PEG block at 3.83 ppm. Thus, ^1^H NMR analysis revealed a number-average MW (*M*_n_^NMR^) of 6.8 kDa for **2**. Moreover, according to GPC analysis, **2** has a *M*_n_^GPC^ of 6.3 kDa with a *Đ*^GPC^ of 1.09, relative to linear polystyrene standards. The narrow and mono-modal GPC peak of **2** also indicates a well-control diblock formation as a result of the living ROP processing.

**Figure 7:**
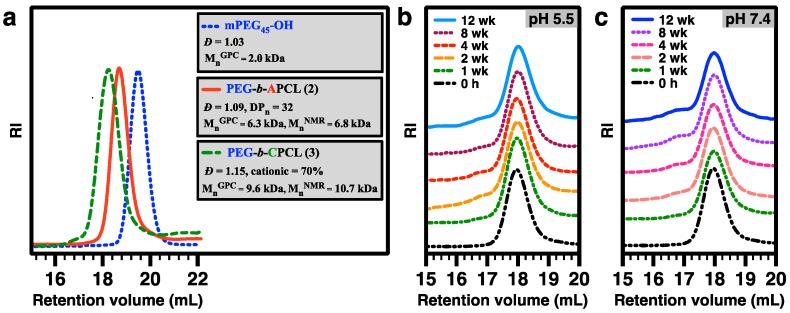
(**a**) GPC characterization of mPEG_45_-OH as the ROP macroinitiator, PEG-*b*-APCL (**2**), and PEG-*b*-CPCL-70 (**3**) with DMF as eluent. (**b**) GPC curves of **3** after incubation at pH 5.5 PBS buffer at 37 °C. (**c**) GPC curves of 3 after incubation at pH 7.4 PBS buffer at 37 °C.

Subsequently, UV-induced thiol-ene functionalization of **2** was conducted in chloroform at room temperature under UV irradiation (λ_max_ = 365 nm) for 40 min, using DEAET as the functionalization agent and DMPA as the photoinitiator ([ene from **2**]_0_:[SH from DEAET]_0_:[DMPA]_0_ = 1:1.1:0.2). The resulting diblock copolymer **3**, i.e., PEG_45_-*b*-CPCL_32_-70 (*M*_n_^NMR^ = 10.7 kDa), has 70 mol % of tertiary amine groups relative to CL-based repeating units, according to ^1^H NMR analysis based upon quantification of resonance intensities of characteristic methylene protons of the amine moieties at ~3.0–3.4 ppm and the remaining allyl protons at 5.02 and 5.74 ppm relative to the C*H*_2_OCO protons of the APCL block at 4.40 ppm (Figure 4). GPC analysis showed a *M*_n_^GPC^ of 9.6 kDa with a *Đ*^GPC^ of 1.15 for **3**. It should be noted that the low *Đ* of **3** is preferred for gene delivery applications, because polymers with high *Đ* values may potentially lead to nanocomplexes with broad size distributions and consequently less controlled biophysical and biomedical properties. GPC curves of mPEG_45_-OH macroinitiator, 2, and 3 are shown in Figure 7a.

**Figure 8:**
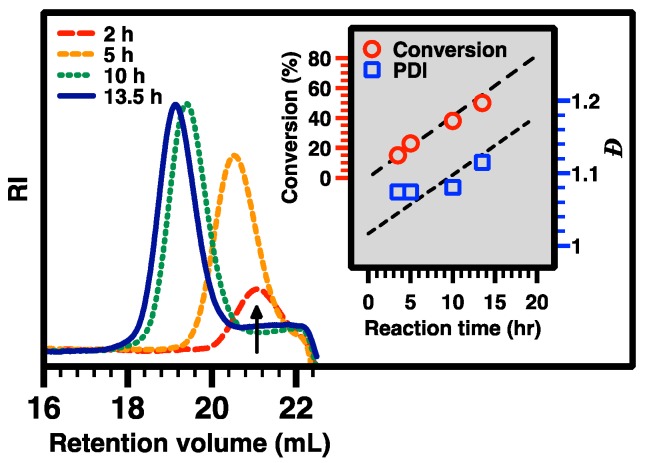
GPC curve evolution during the ROP process to form APCL. A shift toward higher molecular weight was observed, showing the progress of the ROP reaction. The inserted graph represents the conversion of reaction (by ^1^H NMR analysis) and *Đ*^GPC^ (i.e., PDI) of APCL vs. reaction time.

CPCL_35_-50 and CPCL_35_-95 (**5**, *DP*_n_ of 35, with 50 mol % and 95 mol % of tertiary amine groups relative to CL-based repeat units, respectively) were synthesized using the same method for the preparation of **3**, except that BnOH was used as the initiator in the first ROP step (Scheme 2). Allyl-functionalized CL monomer **1** was polymerized via ROP with TBD as organocatalyst and BnOH as initiator ([**1**]_0_:[BnOH]_0_:[TBD]_0_ = 70:1:5; at room temperature for 13.5 h, in dry toluene; ~50% overall conversion of **1**). The chemical structure of the resulting APCL (i.e., **4**) was verified via 500 MHz ^1^H NMR analysis in DMSO-D_6_ (Figure 5). The DP_n_ of 35 was determined based on the comparison of resonance intensity of C*H*_2_OCO protons of PCL block at ~4.0 ppm and terminal aromatic protons at ~7.2 ppm. Accordingly, the number-average molecular weight (M_n_^NMR^) of 5.3 kDa was obtained for **4**. GPC analysis revealed that a M_n_^GPC^ of 3.5 kDa and a *Đ*^GPC^ of 1.11 for **4**, relative to linear polystyrene standards (Figure 8). With the progress of ROP, GPC curves of the resulting polymer species shifted to the high MW side (i.e., left) while maintaining low *Đ*^GPC^ values, suggesting a living polymerization behavior. The low-MW tails of the GPC curves might indicate the minor formation of oligomer presumably due to a trace amount of impurities which affected the ROP process. Nevertheless, the oligomer can be removed through the work-up procedure. Following the same procedure to synthesize **3**, CPCL-50 and CPCL-95 (both referred as **5**) were prepared by UV-induced thiol-ene functionalization of **4** with DEAET at room temperature, with [ene from **4**]_0_:[SH from DEAET]_0_:[DMPA]_0_ of 1:0.7:0.2 (1 h) and 1:3:0.4 (3 h), respectively. Conversion of the allyl groups for CPLAs **5** was determined by ^1^H NMR analysis (Figure 6), using the approach the same as the quantification of the functionalization extent of **3**.

### 3.2. Hydrolytic Stability of PEG-b-CPCL-70 (***3***)

As compared with PLA, PCL as a biodegradable polyester has very slow hydrolytic degradation, and literature reports that it takes more than a year for either bulk or nanostructured PCL to start showing major degradations under aqueous environments at 37 °C [38], although the degradation of PCL becomes faster at elevated temperatures [39,40]. The hydrolytic stability of PCL relative to PLA is among the key considerations for us to design CPCL-based polymers for gene delivery applications. Before performing a hydrolytic stability study for CPCL-based polymers, their aqueous solubility (Table 1) was probed at the concentration of 1.0 mg/mL in 25 mM PBS buffer at 37 °C and physiologically relevant pH values of 7.4 (representative pH for human blood) and 5.5 (representative pH for endosomes). PEG_45_-*b*-CPCL_32_-70 showed aqueous solubility at both pHs. On the other hand, CPCL_35_-50, and CPCL_35_-95 were only soluble at pH 5.5; they formed cloudy dispersions at pH 7.4 with the assistance of sonication, but polymers precipitated a few days later. The pH-dependent solubility of CPCLs can be ascribed to the protonation of the amine group at lower pH that favors a dissolving process. The incorporation of the hydrophilic PEG block with CPCL leads to higher and less pH-dependent solubility of the resulting diblock copolymer relative to CPCLs. Therefore, the subsequent hydrolytic stability study was focused on PEG_45_-*b*-CPCL_32_-70 (**3**).

Hydrolytic stability of **3** was investigated over a period of 12 weeks at 25 mM PBS buffers of pH 5.5 and 7.4 at 37 °C, based on GPC monitoring of the incubated solutions (Figure 7b,c). GPC results showed similar hydrolytic stability of **3** at both pH 5.5 and 7.4. Throughout the period, the polymer peak maintained its original position with gradually appearing shoulders. The shoulder at the low-MW side was less significant, indicating a quite slow hydrolytic degradation. The shoulder at the high-MW side became more noticeable after the first week of incubation, presumably because of the minor occurrence of intermolecular linking reactions of CPCL blocks via the remaining allyl groups. In contrast to the relatively fast degradation of CPLAs [25], the remarkable hydrolytic stability of **3** can be attributed to its significantly lower ester group density on the more hydrophobic aliphatic polyester backbone.

### 3.3. Nanocomplex Formation and Characterization

**Figure 9:**
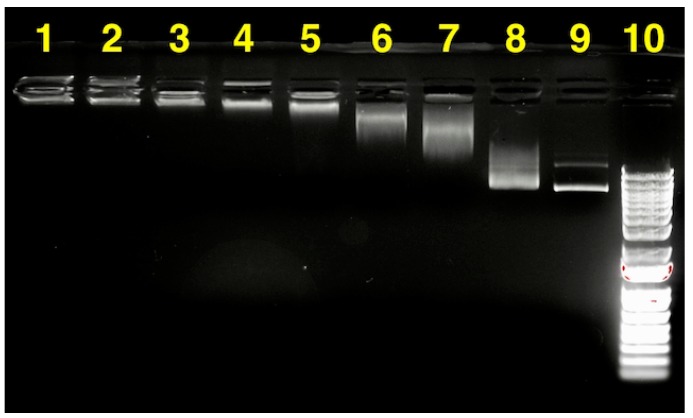
Agarose gel electrophoresis of polymer:pDNA nanocomplexes. Lane assignments are as follows: (1)–(8) PEG_45_-*b*-CPCL_32_-70:pCMV-EGFP pDNA nanocomplexes at mass ratios of (1) 1000:1, (2) 500:1, (3) 200:1, (4) 100:1, (5) 50:1, (6) 20:1, (7) 10:1, (8) 1:1; (9) pDNA, and (10) molecular weight marker (DNA ladder). For the polymer:pDNA mass ratio of 1:1, N/P is 0.71. The concentration of pDNA in each well is 8 μg/mL.

Gel electrophoresis is a direct approach to assess the stability of pDNA in a gene delivery formulation. In order to evaluate the appropriate polymer:pDNA mass ratio for the nanocomplex formation (N/P = 0.71 is equivalent to polymer:pDNA mass ratio of 1:1), agarose gel electrophoresis was implemented to visualize how much PEG_45_-*b*-CPCL_32_-70 is needed to complex with pCMV-EGFP pDNA (Figure 9). At a 1:1 mass ratio of polymer:pDNA (lane 8), most of the pDNA was not complexed and showed migration similar to naked pDNA (lane 9). At mass ratios of 10:1 (lane 7) and 20:1 (lane 6), the migration of pDNA in the electric field was still noticeable, evidently slower than naked pDNA, indicating that free pDNA was absent, but the electrostatic interaction of pDNA with polymer was relatively weak. It was observed that at a polymer:pDNA mass ratio of 50:1 (lane 5), migration of pDNA was significantly retarded, indicating a high level of complexation of PEG_45_-*b*-CPCL_32_-70 with pCMV-EGFP pDNA. Further increase in the mass ratio (100:1 or higher; lanes 1–4) resulted in an even slower migration of pDNA, indicating the stronger electrostatic interaction of pDNA with the polymer.

**Figure 10:**
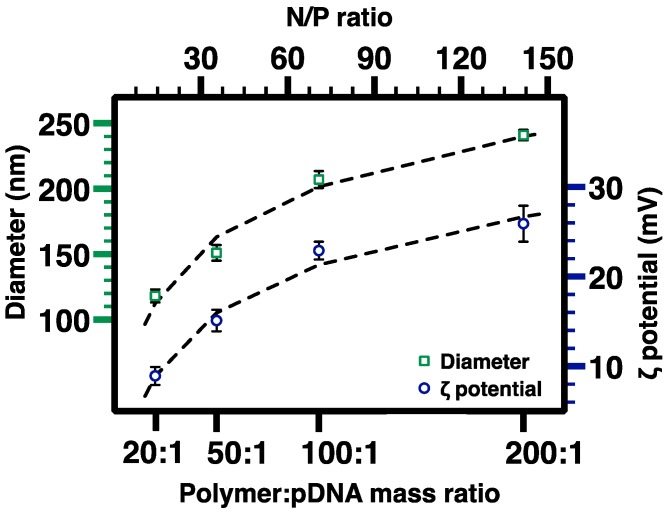
DLS measurement of number-average hydrodynamic diameter (*D*_h,N_) and zeta potential (ζ) of nanocomplexes at various PEG-*b*-CPCL-70:pDNA mass and N/P ratios. The error bars represent the standard deviation from 3 independent measurements.

The surface charge and hydrodynamic size of the nanocomplexes in water were further investigated by dynamic light scattering (DLS) analysis. As shown in Figure 10, the number-average hydrodynamic diameter (*D*_h,N_) of nanocomplexes increased with the increase in polymer:pDNA mass ratio in the range of 20:1 (*D*_h,N_ = 118 ± 5 nm) to 200:1 (*D*_h,N_ = 240 ± 4 nm). Such tunable sizes of the nanocomplexes may be helpful to optimize their gene delivery efficacy because gene transfer studies using parenchymal liver cells as the model cell line suggested that the diameter of sinusoidal fenestrae exerts a size limit for effective vectors [41,42]. Because PEG_45_-*b*-CPCL_32_-70 is positively charged, zeta potential (ζ) of nanocomplexes also increased with the increase in polymer:pDNA mass ratio in the range of 20:1 (ζ = 9 ± 1 mV) to 200:1 (ζ = 26 ± 2 mV). The significant ζ values observed for nanocomplexes at high polymer:pDNA mass ratios can promote their colloidal stability, which is important for gene delivery application.

**Figure 11:**
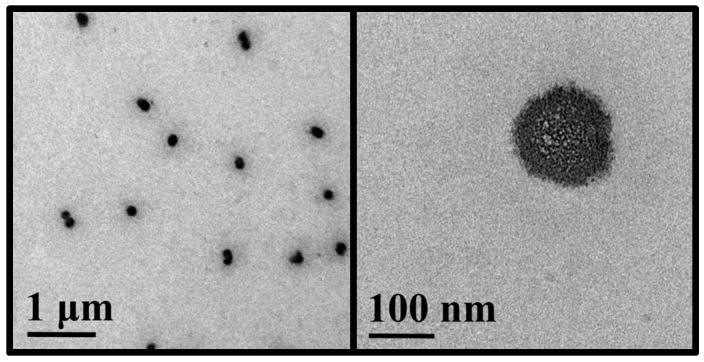
TEM images of PEG-*b*-CPCL-70:pDNA nanocomplexes at 200:1 mass ratio (N/P = 142). The low magnification TEM image (**left**) shows a narrow size distribution of nanosized nanocomplexes, and the high magnification TEM image (**right**) shows the nanostructural details of a nanocomplex.

**Figure 12:**
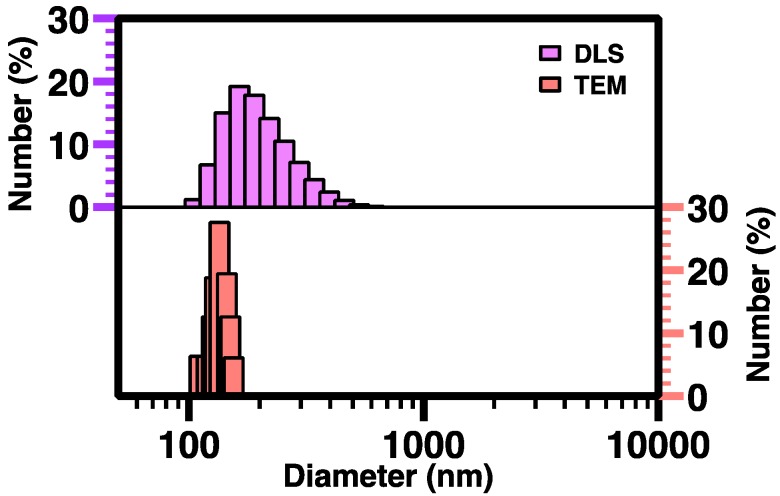
The number-average size distribution of PEG-*b*-CPCL-70:pDNA nanocomplexes at 200:1 mass ratio from DLS measurement (top) and TEM image analysis (bottom).

The morphology and size of nanocomplexes were investigated with transmission electron microscopy according to our previously published sample preparation method (TEM; Figure 11) [43]. The polymer:pDNA ratio of 200:1 for nanocomplexes was selected for TEM characterization, and the corresponding TEM images showed their rough spherical morphologies, with a number-average surface diameter of 135 ± 17 nm. TEM provided a smaller average size and narrower size distribution of nanocomplexes than DLS for the same nanocomplexes (Figure 12) because the nanocomplexes dried and considerably shrank on the TEM grid while DLS gave hydrodynamic size distribution.

### 3.4. pDNA Release and Serum Stability of Nanocomplexes

It is important to assess whether the nanocomplexes can provide protection for pDNA and also allow the release of pDNA in biological conditions. Because direct observation of these phenomena under cellular environments is more difficult, the release of pDNA from the nanocomplexes in aqueous solutions in the presence of heparin (a model anionic polyelectrolyte biopolymer) and the serum stability of the nanocomplexes were probed. Agarose gel electrophoresis was utilized in pDNA analysis and nanocomplexes prepared at polymer:pDNA mass ratio of 200:1 (N/P = 142) were employed in each study.

**Figure 13:**
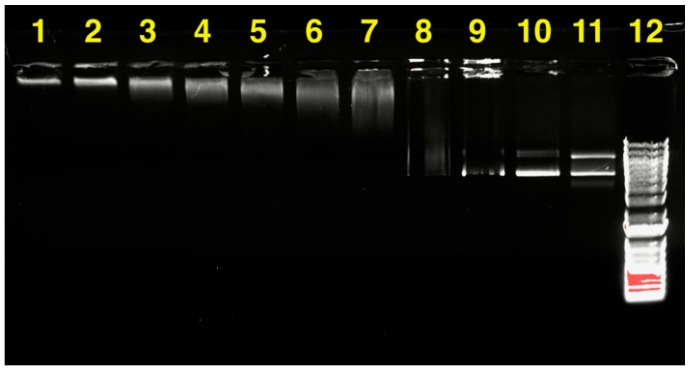
pDNA release from PEG-*b*-CPCL-70:pDNA nanocomplexes at a 200:1 mass ratio (N/P = 142) by increasing polyanionic heparin. The nanocomplexes were incubated for 1 h at 37 °C with different amounts of heparin. (1)–(10) nanocomplex:heparin at mass ratios of (1) 1:0, (2) 1:0.001, (3) 1:0.002, (4) 1:0.004 (5) 1:0.006, (6) 1:0.008, and (7) 1:0.01, (8) 1:0.02, (9) 1:0.04, (10) 1:0.06, (11) pDNA, and (12) MW marker (DNA ladder).

Heparin, as an anionic polyelectrolyte, is able to break the nanocomplex formed from PEG-*b*-CPCL-70 and pDNA. In this study, various amounts of heparin were used to compete with PEG-*b*-CPCL-70 until the pDNA was released from the nanocomplex. At a fixed polymer:pDNA mass ratio of 200:1, which ensured nanocomplex formation, heparin solutions were added gradually, and the mixed solutions were obtained with vortex stirring. The solutions were then incubated at 37 °C for 1 h, followed by running gel electrophoresis to evaluate the unbinding behavior. Figure 13 illustrates the UV visualized image of gel electrophoresis stained by ethidium bromide. It is observed that, with the increase in the heparin mass, the pDNA started to unbind form the nanocomplex matrix. This can be presumably attributed to electrostatic interaction between heparin and PEG-*b*-CPCL-70, which are negative and positive polyelectrolytes, respectively. An incremental increase in the amount of heparin provided more negative charge such that it could overcompensate the existing positive charge in the nanocomplex. Consequently, pDNA species were not bound to any cationic domain and able to be released completely. From lane 3 to 9, pDNA species were gradually able to drift down the in the electric field until in lane 10, and they were completely released as they gave the same spot lines as naked pDNA in lane 11. Although heparin-triggered pDNA release is not cell-realistic and there are many kinds of anionic species under cellular environments, the above result demonstrates that pDNA can be released from the nanocomplexes via electrostatic interactions.

**Figure 14:**
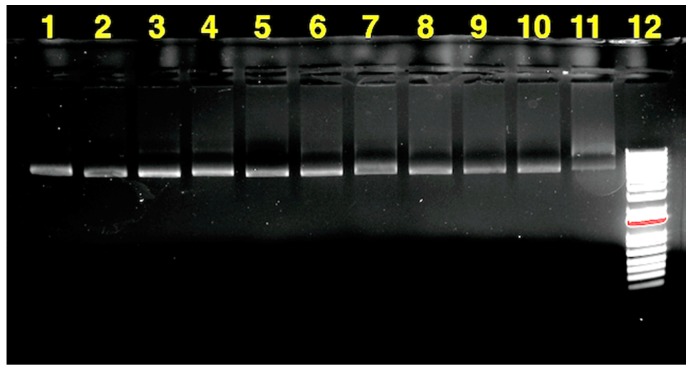
Serum stability studies of PEG-*b*-CPCL-70:pDNA nanocomplexes at 200:1 mass ratio at various time points. Time points were assigned to each lane as follows: (1) 0 min, (2) 5 min, (3) 15 min, (4) 30 min, (5) 45 min, (6) 1 h, (7) 2 h, (8) 3 h, (9) 4 h, (10) 5 h, (11) 24 h, and (12) molecular weight marker (DNA ladder).

Serum endonuclease is capable of fragmenting unprotected pDNA during in vivo transport. One of the most important features of suitable polymeric gene delivery systems is to protect pDNA formulations in the serum environment. Unprotected pDNA can undergo a fast break down in time scales of less than an hour due to serum endonuclease [36]. The shielding feature of the PEG-*b*-CPCL-70 polymeric gene delivery system for pDNA against FBS was investigated directly with agarose gel electrophoresis (Figure 14). Accordingly, 300 μL of polymer:pDNA nanocomplexes at a 200:1 mass ratio (N/P = 142) was combined with 10% FBS and incubated at 37 °C for 24 h. At various time points, aliquots of 25 μL were taken and frozen immediately to stop the serum degradation activity. Prior to agarose gel electrophoresis, the samples were treated with 4 μL (1 mg/mL) polyanionic heparin to enable pDNA to migrate in gel electrophoresis. Intact pDNA species demonstrate similar migration and ethidium staining (lanes 2 to 10 in Figure 14) compared to the pDNA in lane 1 without incubation. Whereas, after 24 h of incubation, the pDNA in lane 11 was almost entirely degraded to smaller oligonucleotides.

## 4. Conclusions

In summary, well-defined cationic PEG-*b*-CPCL was studied as a potential PEGylated non-viral vector for pDNA delivery. It was synthesized by ROP of ACL using PEO_45_-OH macroinitiator, followed by thiol-ene functionalization of the resulting diblock PEG-*b*-APCL. Its chemical structure was confirmed by ^1^H NMR and GPC analysis. PEG-*b*-CPCL exhibited significant hydrolytic stability and did not show a major occurrence of hydrolytic degradation in incubation for 12 weeks. Relative to CPCLs with pH-dependent solubility, PEG-*b*-CPCL can provide solubility over a wide range of pH. It can complex with pDNA to form positively surface-charged spherical nanoparticles, with both hydrodynamic size and surface charge increasing with the increase in the polymer:pDNA ratio. The nanocomplexes can protect pDNA and also allow the release of pDNA. PEG-*b*-CPCL possesses a remarkable combination of biomedically relevant properties (i.e., significant hydrolytic stability, biodegradability, and complexation ability with pDNA). The above results encourage further studies to investigate PEG-*b*-CPCL as a promising synthetic vector for gene delivery applications.

## Figures and Tables

**Table 1:** Solubility of CPCLs and PEG-*b*-CPCL (**3**) in 25 mM PBS buffer at pH 5.5 and 7.4 at 37 °C.

Polymer	Solubility in	
	PBS pH 5.5	PBS pH 7.4
PEG-*b*-CPCL-70 (**3**)	√	√
CPCL-50	√	×
CPCL-95	√	×

√: soluble, ×: insoluble
